# Household Point-of-Use Faucet Filters for Lead Removal: Field Performance and User Experiences

**DOI:** 10.1021/acsestwater.4c01257

**Published:** 2025-05-09

**Authors:** Jeannie M. Purchase, Chantaly Villalona, Adrienne Katner, Kelsey Pieper, Marc Edwards

**Affiliations:** † 1757 Virginia Tech, Department of Civil and Environmental Engineering, Blacksburg, Virginia 24061, United States; ‡ 12258 Louisiana State University Health Science Center, Department of Environmental and Occupational Health Sciences, New Orleans, Louisiana 70112, United States; § 1848 Northeastern University, Department of Civil and Environmental Engineering, Boston, Massachusetts 02115, United States

**Keywords:** point of use lead filters, particulate lead, iron clogging, consumer experience

## Abstract

Lead certified point of use (POU) faucet filters were field tested in two Louisiana cities in a total of 21 occupied homes during normal use and in two unoccupied homes with very high risk of water lead (Pb) contamination. In an unoccupied home with a 28 m lead service line (LSL) with average lead of 17 μg/L in flushed water, treatment by POU filters generally produced water with <5 μg/L Pb even when tested to 200% of rated capacity. In the unoccupied home with a disturbed LSL, the water had erratic influent particulate lead of 9–3053 μg/L, and the POUs did not consistently produce water with <10 μg/L Pb despite very high percentage removals. When testing POUs in occupied homes known to occasionally have lead over 5 μg/L, POUs always reduced lead to <1 μg/L. In addition, POUs were also effectively removing high levels of iron and manganese present in the water of one city, but this also caused clogging before reaching half of their rated capacity. Laboratory experiments confirmed POU susceptibility to iron clogging.

## Graphical abstract

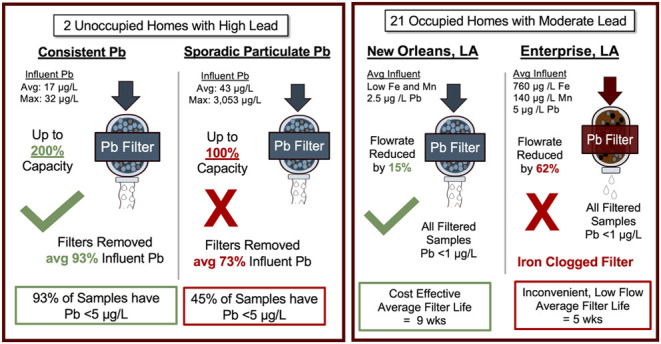

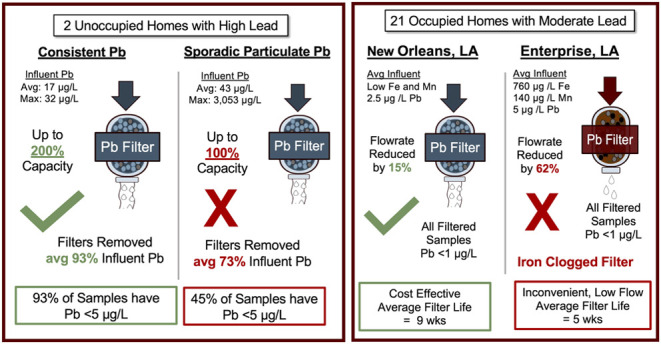

## Introduction

In recent years, point-of-use filters (POU) have been used to reduce lead exposure during water crises, in schools and day care facilities, and after lead service line (LSL) replacement.^1–10^ Filters are often considered more environmentally friendly and less expensive than bottled water.^10–12^ Some small water systems with less than 3300 consumers will soon be allowed to use POU filters as a means of complying with the Lead and Copper Rule (LCR) in place of corrosion control.^13^

A recent literature review highlighted prior research evaluating filter performance.^14^ Over a decade ago it was demonstrated that POU filters removed soluble lead effectively, but they occasionally had problems removing particulate lead.^1^ Follow up work indicated that POU filters were generally very effective at reducing lead levels below <5 μg/L in field studies both in Flint (100%, *n* = 242) homes, and in Newark, NJ (97.5%, *n* = 198).^7,15^ There were a few exceptional cases identified in Newark, NJ (2.5%, *n* = 198) in which filtered water lead levels were as high as 112 μg/L.^14^ Subsequent lab work revealed the POU filters had difficulties removing lead nanoparticles, large lead particles, and sometimes had manufacturing defects that contribute to poor removal.^16–18^ Nonetheless, POUs are generally found to be effective at reducing elevated lead levels even when operated beyond their rated capacity, despite exceptional cases of relatively poor performance.^1,7,8^

As utilities and consumers increasingly rely on POUs to protect public health from lead exposure, there is a need to better manage consumer expectations and understand factors that limit their performance in the field. Premature clogging of filters due to iron and other sediment is an important emerging concern.^9,11,19^ Rouillier et al. recently reported a correlation between the influent iron concentration and the length of time before clogging for gravity fed pitcher filters,^11^ and further demonstrated that above certain iron thresholds bottled water will be more cost-effective. Similar factors that limit consumer adoption of pressurized POU faucet filters are also deserving of increased attention.^20^

Here, we evaluate various aspects of faucet mount POU filter performance in longitudinal field studies. Specific goals were to (1) sample water without filtration to characterize lead levels as a function of water flushing (control condition), (2) monitor POU faucet filter performance under extreme lead exposure risk scenarios in two unoccupied homes for 200% of each filters rated capacity, and (3) monitor performance in occupied homes during routine use comparing a situation with low iron to that with high iron, and (4) verify problems with iron clogging in laboratory experiments.

## Methods

### Study Area and Sampling Efforts

This study was conducted in New Orleans (NOLA) and Enterprise, LA (ELA) homes identified with water lead concerns. NOLA represents a diverse urban community with a high proportion of low-income residents.^21^ In NOLA, the municipal drinking water supply meets regulatory requirements for lead, but about 5% of homes still exceed the 15 μg/L EPA action level in effect at the time this article was written.^22^ We previously illustrated that NOLA samples collected after about 1 min of flushing often have higher lead than first draw.^22^ Elevated lead after partial replacement of LSLs in NOLA is also an important concern despite utility dosing of a polyphosphate corrosion control product.·

In ELA, our prior sampling revealed that 41% of homes had lead above the 15 μg/L EPA action level. This was not surprising due to a lack of phosphate corrosion control and pHs that dropped below 7 during stagnation in many homes. Moreover, 71% of sampled homes exceeded the aesthetic standard for iron and 94% exceeded the aesthetic standard for manganese.^23^ There are no lead service lines in ELA so the source of contamination is leaded solder and leaded brass. This is a very rural community with only about 250 residents that cannot afford centralized removal of iron and manganese from the water.^24^ ELA is representative of a small system which might try to use POU filters for LCR compliance.

We evaluated POU performance using two distinct sampling efforts. The first was in two unoccupied homes that were challenged by running filters to 200% of capacity in situations with serious water lead hazards. The second sampling effort was in occupied NOLA and ELA homes to measure lead removal rates, flow rates, and user perceptions under routine use.

### Unoccupied Home Study

We sampled in two NOLA homes that were unoccupied during testing, in order to evaluate two practically important and high water lead risk scenarios, without endangering consumers when filters were used beyond manufacturers recommendations for influent lead levels and filter lifetime. A coauthor of this paper (Katner) owned one of these homes and knew the owner of another. Home A was estimated to have an extremely long 92 ft (28 m) long LSL with documented sustained water lead release (average 17 μg/L) problems.^23^ Home B was used to test a 6 ft (1.8 m) long LSL that was extracted from service and which had been severely disturbed physically. Such lead service line disturbances occur in practice after partial lead service line replacements and other construction.

#### Rig Design and Flow Sequence

We designed a semiautomated sampling rig to determine the effectiveness of filtration through POU filters (Supporting Information Figures S1 and S2). An NSF-ANSI 53 lead faucet filter certified to a 5 μg/L standard was tested in duplicate in each home. The POU selected used carbon-block technology and had a 100-gallon capacity. Our prior testing demonstrated that this brand consistently reduced lead to less than 15 μg/L for up to 200% capacity in a laboratory setting.^16^ Flow was controlled using solenoids, valves, and timers that were powered by a DC rechargeable 12 V 35 Amp hour battery. The flow sequence through each lead pipe was a 7.5-h stagnation event, followed by 0.5 h flow, which was repeated three times daily. Flow was for 20–30 min at 0.3–0.5 gallons per minute (gpm) to achieve a target of 10-gallons per event (i.e, 10% of treatment capacity). To prevent water damage to property the entire apparatus was housed in a secondary container equipped with an emergency shut-off valve triggered by leak sensors. We installed the rig in Home A by connecting it directly to the kitchen tap, whereas in Home B the disturbed lead pipe was connected to a sink to provide influent flow and the pipe effluent was connected to the sampling apparatus. The rigs were operated in both homes for 20 days to achieve 200% of the POU rated capacity. For unit consistency, the unoccupied home study reports volumes in gallons for direct comparison to units of the manufacturer’s published filter capacity.

#### Stagnation Sampling

Every drop of water passing through each LSL for 20 days was collected and sampled. The first flow event through the LSL each day was directed though one of the POU filters and into a reservoir dedicated to that event. The second flow event each day did the same for water treated by the duplicate POU filter with flow directed into a reservoir dedicated to that event. The third flow event went directly into a third reservoir with no filtration as a control. The control sample is representative of the untreated water that was influent to the filters. Prior to each sampling event the sampling reservoirs were dosed with 50 g of Alpha Chemicals food-grade citric acid. QA/QC demonstrated that this safely reduced pH to <3.0 and prevented soluble lead sorption to the walls of containers. After rigorously mixing the acidified reservoir to homogeneously suspend any undissolved lead particulates, we collected an aliquot from the reservoir in a 250 mL container.

#### Profile Sampling

A special profile sampling using multiple bottles instead of the reservoir, was collected from Home A on days 0, 10, and 20 and Home B on days 0, 8, 14, and 20. After the 7.5 h stagnation, flow was initiated and 15 one liter samples were manually filled in sequence, followed by 250 mL grab samples after a 5, 7, and 9 gallons had been flushed (Figure 1). All water not collected in a bottle was directed into the sampling reservoirs. After stirring the acidified samples as before, a 250 mL composite sample from the reservoir was collected. To determine the fraction of particulate lead, 10 mL aliquots were immediately collected from samples 1, 3, 5, 7, 11, 15, and 17 after passage through a 0.45 μm pore size filter.^25,31^ After returning to the lab, all samples were acidified with 2% nitric acid and digested for a minimum of 16 h prior to analysis on a Thermo Electron iCAP RQ Inductively Coupled Plasma Mass Spectrometer (ICP-MS) per standard method 200.7 and 200.88.^25^ If iron was present, samples underwent additional digestion prior to analysis using 2% hydroxylamine hydrochloride and a heated digestion (50 °C) for 24 h.

**Figure 1:**
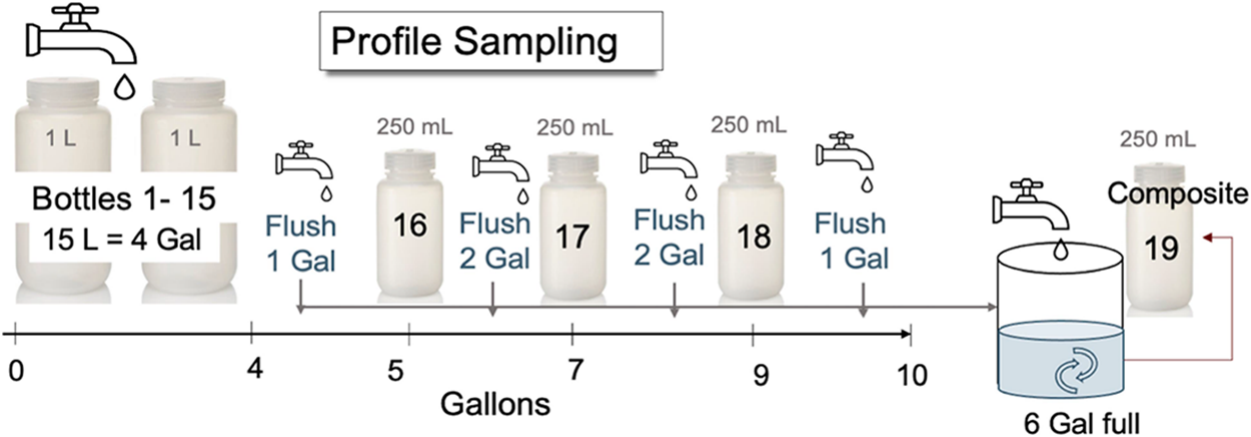
Special profile sampling using a sequence of bottles after 7.5 h stagnation.

### Consumer Testing in Normal Field Use

We provided faucet POUs to residents in NOLA and ELA with known lead in water problems to monitor long-term performance of POUs and user behaviors. These consumers were recruited from a prior study, in which we had previously sampled drinking water from 376 homes in NOLA^22^ and 17 homes^23^ in ELA. All participants with >5 μg/L lead in first draw or flushed samples were invited to participate in this study. Our previous experience suggested that filters in ELA water with high iron and manganese might have problems with clogging.

The research team visited interested participants to install the POU filters and train participants in their proper use (e.g., not filtering hot water, how to change cartridge). Only homes with a kitchen tap design suitable for use of a POU were considered (e.g., removable aerators). One home was excluded on this basis. In addition, participants were given a sampling kit that contained sampling bottles, instructions, reminder cards, a stopwatch, a permanent marker, and prepaid return postage.

This human subject experiment was reviewed and approved by the Virginia Tech IRB board (17-541). The study protocol, informed consent procedures and data collection instruments were reviewed and approved by the Louisiana State University- Health Institutional Review Board (IRB # 887 and 10069).

Participants in the study were asked to use the POU as per their normal daily routine and to collect a weekly first draw sample for 12 weeks unless (1) the POU reached its capacity before 12 weeks as indicated by its built-in indicator light turning yellow/red or (2) the participant felt that the POU was clogged (i.e., filtering water was taking too long). It is important to note that NSF/ANSI 42 and 53 defines POU clogging as the time at which the initial flow rate is reduced by 75%.^26,27^ While the POU had a design flow rate of 1.9 L per minute (lpm) at 60 psi, the initial flow rate will fluctuate with varying water pressures in homes.

The night before sampling, participants were instructed to let the water remain stagnant overnight for at least 6 h. Thereafter, participants collected a 250 mL unfiltered first draw sample. For comparison, the next night, this process was repeated but participants collected a 250 mL filtered first draw sample from the POU. In addition, participants were asked to measure the time required to fill the bottle. If participants felt their filter was clogged, they were asked to repeat the filtered first draw sampling one additional time. As before samples were acidified and digested with 2% nitric acid and 2% hydroxylamine hydrochloride (as needed) prior to analysis on the ICP-MS.

Participant and household information were collected via interviews and observations during a home visit to install the filters, at which time study consent forms were signed and collected. Participants were asked to complete a survey when the research team visited to install the POU and at the end of the study. Once the lifetime of the filter had been reached or the resident discontinued use of the filter, residents were surveyed to assess their use of the filter, perceptions of the quality of their filtered water, ongoing support or information needs, problems encountered during POU filter testing, knowledge of filter maintenance, their likelihood of continuing POU use, and reasons for discontinuing use.

### Iron Flow Reduction Laboratory Study

We conducted a laboratory experiment to validate consumer belief that brown and red water (i.e., iron) was clogging the filters in ELA. We selected four brands of faucet POUs for analysis and tested performance in duplicate. We exposed POUs to the standard National Sanitation Foundation test water but with increasing iron concentrations: 0, 150, 300, 600, 1000, 2000, 4000, and 5000 μg/L Fe added as FeCl_3_ during the experiment. All of the iron was particulate as described by filtration through a 0.45 μm pore size filter.

We used the automated faucet rig described in Purchase et al., which was powered by a booster pump and had a water pressure range between 35 and 45 psi.^16^ Each filter was preconditioned by flushing iron free water for 5 min prior to testing. During testing, we filtered 11 L (3 gal) of each test water through the POU followed by 11 L of deionized water. We measured the length of time required to fill a 250 mL bottle during the deionized water flush. We continued to expose the POU to this test water and repeated this process until the filtration time to fill the 250 mL bottle doubled.

## Results

### Controlled Testing in Unoccupied Homes

#### Home A with Long LSL and Sustained Lead

The unoccupied home with sustained high-water lead had a very long lead service line that produced water with average lead (10-gallon composite) of 10–24 μg/L (Figure 2B) during the 20-day study. During the 10-gallon flushed profiles taken on Day 0, 10, and 20, the lead from the tap without filtration fluctuated between 5.6 and 31.3 μg/L and the average percentage particulate lead was 15–21% of the total lead (Figure 2A). As water flowed from the tap after stagnation events, the lead concentrations in the control sample without a filter peaked after flushing in bottles 15–17 (i.e., 4–7 gallons of total flow), consistent with continuous flushing of loose lead sediment from the long service line. Except for the first 6 L of the Day 0 profile, lead levels were always >10 μg/L even after flushing >8 min (10-gallons). Clearly, the NOLA public health recommendations to flush the tap 30 s–2 min, to reduce lead exposure in water used for cooking or drinking, would not have been effective in this home, which was worst case for all homes we sampled in the city.^22^ As the study continued the lead in the unfiltered control samples increased slightly from Day 0 up to Day 20. This rising level of lead might reflect the relatively low water use of 30 gallons per day during this study, allowing loose lead deposits to accumulate somewhat in the service line, compared to the high flow of 120 gallons per day^28^ typical water use when this home was occupied.

**Figure 2:**
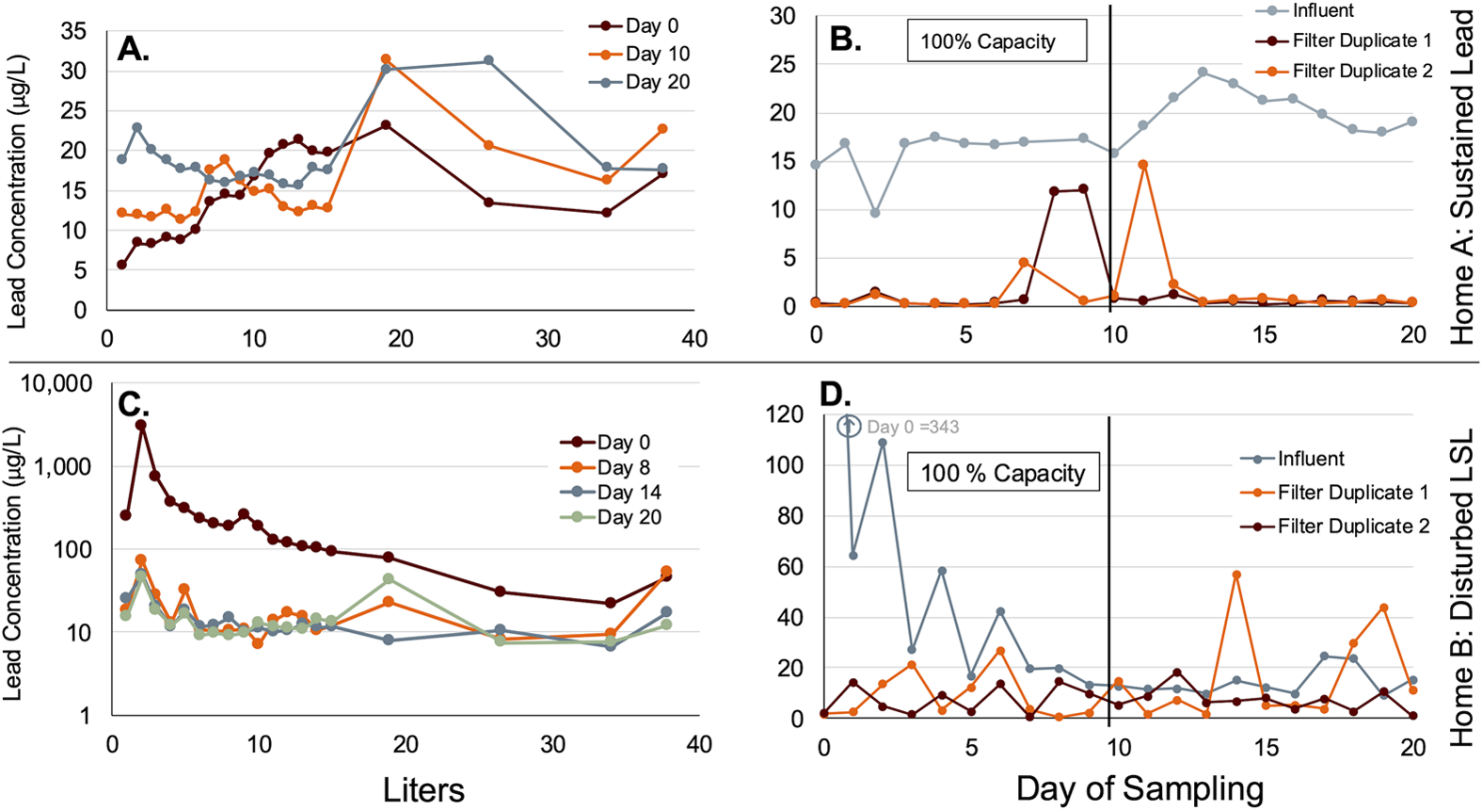
Unoccupied Home profile sampling and filter performance over time. (A) Lead profiles of Home A with Sustained Lead. (B) Duplicate POU filtered lead concentrations for Home A with Sustained Lead. (C) Lead profiles of Home B with Disturbed LSL. (D) Duplicate POU filtered lead concentrations for Home B with Disturbed LSL.

When the faucet filters treated that water, they consistently produced water with less than 5 μg/L lead, except for 3 exceptional samples which had >10 μg/L lead (Figure 2B). Filter duplicate 1 had lead levels below 2 μg/L and removal efficiencies between 85.2 and 99.6%, except for Day 8 and 9 (composites of cumulative gallons 80–100 of treated water) which had effluent lead of 12 μg/L that occurred before reaching the filter’s 100-gal capacity. The filtered lead concentrations when tested beyond the rated capacity were always <2 μg/L. Duplicate 2 always had effluent lead levels below 5 μg/L and removals of 73.8–99.9%, except for one sample with 15 μg/L on Day 11 at a point just 10% beyond the rated capacity.

#### Home B with Disturbed LSL

The four 38 L (10-gallon) profiles of unfiltered water in the unoccupied home with a disturbed LSL had the highest lead concentrations in the first 4L, after which the concentrations steadily declined (Figure 2C). The lead levels in the profiles always peaked in bottle 2 (2L), which corresponds with the water sitting stagnant in the rig’s disturbed LSL (6 ft long and 1 in diameter). Day 0 had the highest lead levels with the 2 L sample at 3,053 μg/L lead and an average particulate lead of 89% (Figure 2C, Supporting Information Figure S4). In practice, LSLs often release very high levels of lead immediately after a physical disturbance such as this. In this case the lead decreased steadily with flushing even on the first day of use. After a physical disturbance lead levels often tend to decrease with weeks, months or years of flushing and pipe recovery. In this case the profiles collected on Days 8, 14, and 20 had average particulate lead at 36–41% of the total lead, with a lower level between 7 and 55 μg/L lead compared to the much higher levels on Day 0 (Figure 2C). Throughout the entire study the 10-gallon composite lead levels were always between 9 and 344 μg/L.

This very high lead coming out of the disturbed pipe of home B proved challenging for the POUs, as they could not consistently treat the water to levels <10 μg/L. Duplicate filter 1 produced 5 grab samples with effluent lead levels >10 μg/L before reaching the rated capacity of 100 gallons. These high levels of lead occurred on Days 2, 3, 5, 6, and 10 and ranged from 12 to 27 μg/L (Figure 2D). The estimated POU removal efficiencies ranged from 21 to 99%, based on the best available estimate obtained when comparing the unfiltered control condition to the corresponding filtered condition collected 8 h apart. Duplicate filter 2 also had lead levels >10 μg/L in 3 samples with effluent lead of 14–15 μg/L on Days 1, 6, and 8 with removal efficiencies of 25–99%.

Home A with Sustained Lead levels never had filtered water lead concentrations greater than the comparison to the control. However, Home B with the Disturbed LSL had filtered lead exceed the corresponding control profile lead on 4 different days between Day 10 and 20. There are two possible explanations for this surprising result. First, when particulate lead release is semirandom, the control sample might be wildly higher or lower than the actual influent to the filter on the same day. For instance, the Day 14 samples of untreated water had influent lead of 15 μg/L (Figure 2C), whereas the corresponding sample for the filtered water lead later was 57 μg/L when collected 8 h later (Figure 2D). Due to the erratic nature of the lead release, it is possible that the actual influent to the filter that corresponded to that effluent sample, was much higher than indicated during the control sample. Second, effluent from a filter can also be higher than the influent, when previously removed lead particulates are suddenly released from within the filter at semirandom intervals as documented previously by Deshommes et al.^1^

These findings reinforce problems of calculating removal efficiencies when there is semirandom release of discrete particles of lead. Our approach of capturing and analyzing every drop of water minimizes, but does not eliminate, this natural variability. It is hypothetically possible that the POU lead removal efficiencies were always greater than 90% relative to the unknown influent concentration on the day of sample collection.

### Consumer Field Testing of POU Performance in Occupied Homes

Water quality from NOLA and ELA illustrated important differences that could affect filter performance (Figure 3; Supporting Information Tables S1 and S2). Specifically, the 8 NOLA homes had unfiltered water with relatively low levels of lead (Pb), iron (Fe), and manganese (Mn). The unfiltered lead levels were all less than 5 μg/L, except for one sample with 22 μg/L lead. Over 75% of iron concentrations in NOLA unfiltered water, were below 10 μg/L, with a maximum of 57.3 μg/L, and manganese levels were all <1.3 μg/L. None of the NOLA samples exceeded either the EPA Secondary Maximum Contaminant Level (SMCL) for Fe (300 μg/L) and Mn (50 μg/L).

**Figure 3:**
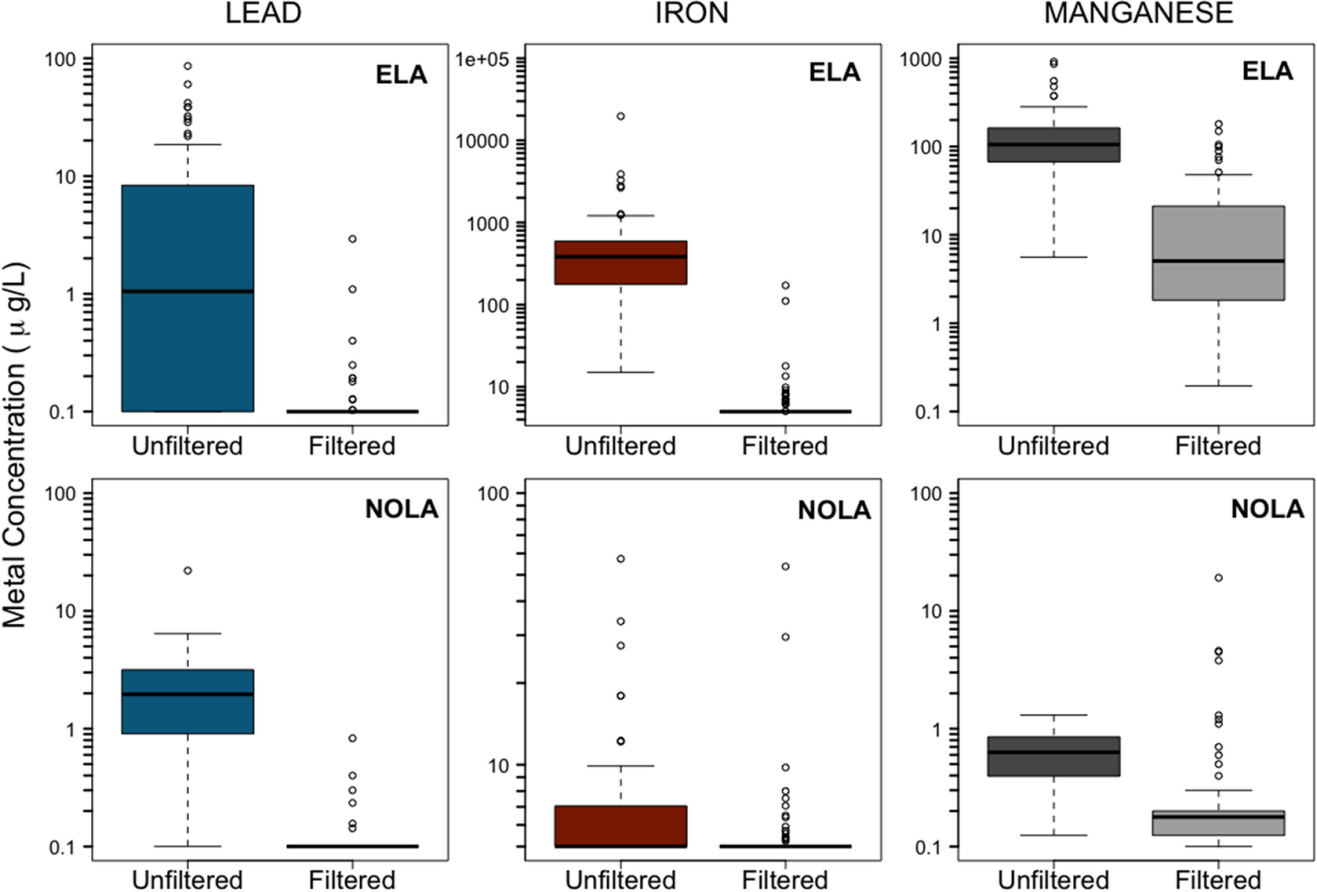
Influent and effluent metal concentrations.

In contrast, 4 out of 13 ELA homes had samples that exceeded the 15 μg/L EPA Action Level for lead at some point in the study with a maximum value of 86 μg/L. The median unfiltered Pb concentration for all ELA homes was only 1 μg/L (Supporting Information Table S2). ELA also had several unfiltered tap samples that exceeded the EPA SMCL for both Fe and Mn. The unfiltered median Fe concentration was 383 μg/L with a maximum of 19,700 μg/L, and the median unfiltered Mn concentration was 106 μg/L with a maximum of 917 μg/L (Supporting Information Table S1).

All filtered lead samples for ELA and NOLA were <0.8 μg/L, iron <171 μg/L, and manganese <180 μg/L. The filters performed well in reducing lead and iron levels in all homes throughout the duration of the study (Figure 2, Supporting Information Tables S1 and S2). However, 10% (*n* = 88) of all filtered samples in 4 ELA homes exceeded the manganese SMCL of 50 μg/L (Supporting Information Tables S1 and S2). The average POU removal efficiencies were 94.8% for Pb, 98% for Fe, and 79% for Mn for the ELA homes, Overall, the filters effectively removed Pb and Fe throughout the test, but the performance in removing Mn was less consistent. NOLA homes always had relatively low Fe and Mn in the influent and filter effluent.

### Consumer Experiences with Clogging

Consumers in our field survey suggested that there was a serious problem with filter clogging when high iron was present. Third party certification testing under guidelines of NSF/ANSI 53 for lead and 42 for Nominal Particulates defines the point of filter clogging as the time at which the initial flow rate is reduced by 75%.^26,27^ The POU filter used in this study has a design flow rate of 1.9 lpm at 60 psi, but the initial flow rate fluctuates with varying ambient water pressures in homes. The initial flow rate for the faucet filters in this study ranged from about 0.9 to 2.5 lpm (Figure 2).

Residents decided when the filter was clogged based on their own judgment. For instance, in one case, a resident decided the filter was clogged when it took 15 s to filter 250 mL (≈1.0 lpm). At another extreme, one resident did not consider a filter to be clogged even when they had to wait >3 min to filter 250 mL of water (<0.08 lpm). On average, the final flow rate for ELA homes at the point consumers replaced the cartridges or abandoned the study, was 0.55 lpm. In the few homes where residents replaced cartridges in NOLA the final flow rate was 1.28 lpm (Figure 4).

**Figure 4:**
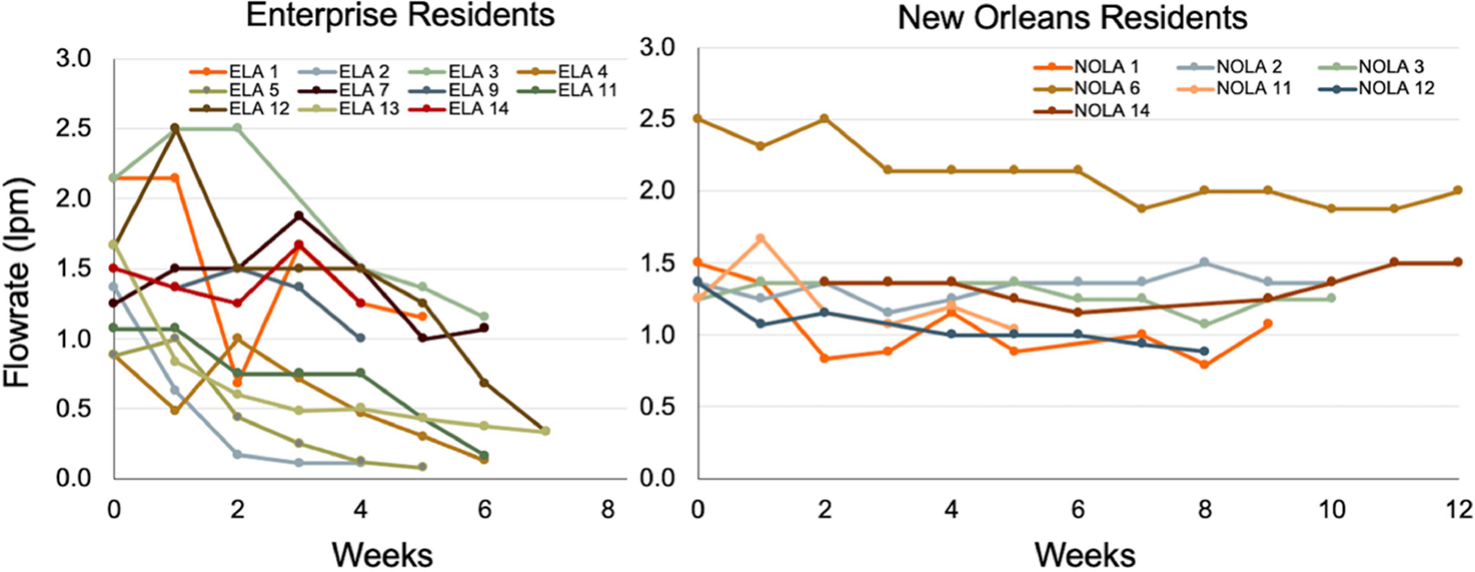
Filter flow rate in NOLA and ELA over time. The POU’s manufactured rated flow rate at 60 psi is 1.9 lpm.

Examining these results from the perspective of percentage reduction from initial flow rates, residents in NOLA with relatively low iron and manganese only saw flow reductions of 16% on average before the end of the study. In ELA, at the point where a resident declared the filter clogged, its average flow was 62% of its initial value. This is lower, but in the range, of the 75% flow reduction clogging criterion for the NSF testing protocol.

The POU filter had a rated capacity of 100 gallons (380 L) or 12 weeks. By this standard none of the filters in ELA reached the filter’s capacity. Most were replaced before 50% of the rated capacity. As a result, these residents would likely need to replace the filter cartridges 2–4 times more frequently than expected based on the rated capacity, making costs of POU use much more expensive.

### Demonstrating the Impact of Iron on Faucet Filter Life

In the field study, we only found a very weak correlation between the average iron concentration in each ELA home and the filter life based on its weeks of use (Figure 5A). Even if iron was completely responsible for the clogging, a weak correlation is not surprising, given the wide variations in water use each day by consumers, and the previously noted variation in how consumers judged that a filter was clogged.

**Figure 5:**
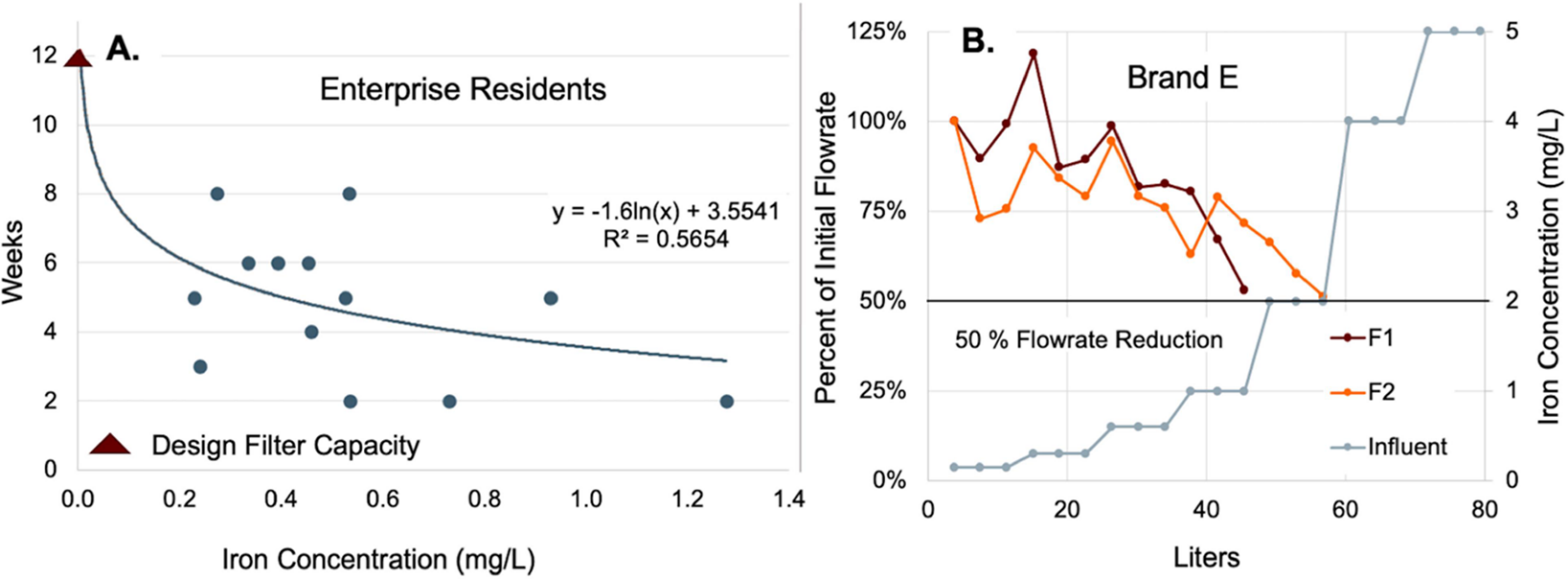
Iron concentration and filter life in the field study at Enterprise homes (left), and representative flow rates in response to increasing iron influent levels in the laboratory clogging study (right).

We conducted a controlled laboratory experiment, that sought to determine if the correlation between iron removal and clogging would improve without the variability introduced by consumer use and judgment. Our study was designed to quickly estimate the mass of iron that would clog the faucet filters to the point of a 50% flow rate reduction (Figure 5B). When testing the POU used in the field study (Brand E), duplicate filter F1 required 23 mg of Fe to reduce the initial flow rate of 1.3 ± 0.3 lpm by 50% whereas duplicate F2 removed 46 mg iron before achieving the same reduction in flow (Figure 5). On this basis it seems clear that even duplicate filters have highly variable susceptibility to clogging. The testing of faucet filter Brands D, F, and K (Supporting Information Figure S5) showed less variability between duplicates in terms of the iron removed at the point of a 50% reduction in initial flow. Specifically, Brand D clogged at 77–92 mg of total iron accumulation, Brand F clogged at 32–47 mg of Fe removal, and Brand K clogged at 62–92 mg of Fe removal. Overall, the laboratory studied confirmed the role of iron in rapid clogging of pressurized POU faucet filters.

## Discussion

This is the first field study to examine lead faucet POU filter performance in two case studies with extreme water lead risks, using an approach in which all flow was rigorously controlled electronically, every drop of water was sampled, and consumers were not endangered. A unique survey was also conducted that examined variability in how consumers assessed clogging problems, with a complementary laboratory study that examined the clogging issue for pressurized POU faucet filters. Other practically important observations are as follows.

### Effects of Flushing to Reduce Lead Levels

The control testing in unoccupied homes, confirmed prior research that the effectiveness of flushing according to available public health guidelines (for 30 s–2 min prior to filter use) is dependent on the situation. In a study conducted by Katner et al. using flushing samples from NOLA homes (Feb 2015–Nov 2016), there was no reduction in lead after 3 min of flushing in 81% (*n* = 372) of homes. Lead was reduced with flushing in 13% of homes but increased in 6% of homes.^22^ Here we profiled the lead concentrations for up to 10 gallons (>8 min flushing) in the home with the long LSL multiple times throughout the 20-day study. Out of the 3 profiles (19 bottles each) collected in the home with sustained lead, the maximum lead concentration was 31.3 μg/L. The lowest flushed sample occurred in the first sample 5.6 μg/L and 91% (*n* = 57) of flushed samples were >10 μg/L. In the home with the disturbed LSL (4 profiles), the lowest flushed concentration was 6.7 μg/L, and 86% (*n* = 76) of flushed samples were >10 μg/L. There was always detectable lead in all the samples even with extended flushing.

### Filter Performance for Lead Reduction

All the filtered samples collected by residents in the occupied home testing in NOLA and ELA homes had lead levels below 1 μg/L. In the ELA homes, the filters removed iron to levels well below the SMCL. However, the POU removal of manganese was inconsistent (27–100%), even though most filtered samples (75%, *n* = 88) had below 25 μg/L Mn (Supporting Information Table S1). The relatively poor removal of manganese was consistent with reports of Carriere et al.^28^ The occasional low removal of Mn did not correlate with the removal of Pb or Fe, consistent with prior work on pitcher filters with high removal efficiencies with co-occurring Fe/Pb waters.^11^ Likewise in Flint, MI, 19% of homes had unfiltered iron above the SMCL of 0.3 mg/L, but this did not affect the removal of lead by the POU.^6^

The faucet POUs in the Home with Sustained Lead performed well in reducing lead levels up to 200% capacity as treated water in the composite samples was <5 μg/L, with only 3 samples >10 μg/L among both filters (400-gallons filtered). In contrast, POUs in the Home with the Disturbed LSL had inconsistent lead removals of 22–99% that decreased over time. Duplicate POUs had 8 out of 20 samples >10 μg/L prior to 100% filter capacity. The poor removal by the POUs in the Home with the Disturbed LSL (treated water >10 μg/L) is consistent with other laboratory and field studies with difficult to treat lead particulates.^7,16–18^

These results reinforce the strengths and weaknesses of remedial flushing and filters. They also point to the importance of managing expectations of consumers for lead removal performance after extreme events such as partial pipe replacements.^11,29–32^ There is a high risk of elevated particulate lead after disturbing lead pipes and the use of POU filters always markedly reduced lead exposure, but not always to below 1, 5, or 10 μg/L.

### Filter Performance from a Consumer’s Perspective

The faucet filter used in these field studies was selected because it had adapters that allowed it to fit most faucets, a bypass valve to test the unfiltered water, and easy installation and removal by residents. Some consumers felt the filter was too big (blocking the sink) for their household sinks, though the filter was about the same size as other commercially available filters. There was only one instance where the filter was incompatible with the existing faucet.^20^

Some residents reported the filter flow rate was too slow even when new. When installed in NOLA and ELA homes the initial flow rate ranged for this POU from 0.68 to 2.5 lpm with an average of 1.35 lpm. Out of 7 filter brands tested in a prior laboratory study the clean filter flow was 1.21 ± 0.23 lpm at 35–45 psi. The POU brand selected for use in this study was the second slowest filter with an average of 1.05 lpm and it did clog quickly in ELA.^16^

### Greater Impact of Clogging

A significant factor that emerged in this study is that clogging with iron can be a major problem for consumers even when using pressurized faucet filters. POU filters might not be suitable in waters with higher turbidity or discolored waters, such as those that contained high levels of iron and manganese described herein. We recently demonstrated that in waters with high iron or high particulates, the use of gravity fed pitcher POUs can be more costly than purchasing store-brand bottled water if they clog.^11^ Similar trade-offs should be evaluated for pressurized faucet filters. It is hypothetically possible that flushing the water before it was applied to the POU faucet filters, or using a whole house prefilter, could reduce the burden of particulates to the faucet POU filter and increase the time before clogging, but that evaluation would require additional research.^20,33^ Another recent study has also reported that consumers were frustrated by the low flow rates associated with POU faucet filters.^34^

## Conclusions

Water lead levels can be reduced by many different strategies. All remediation strategies including (1) distribution of bottled water, (2) remedial flushing to reduce lead levels, (3) installation of lead-certified point-of-use filters, and (4) replacement of leaded plumbing have strengths and limitations.

Our studies in unoccupied high-risk homes showed that flushing did not always satisfactorily reduce lead levels even after >8 min. The presence of high particulate lead after disturbing an LSL, created a severe challenge to faucet POU performance, and the lead levels frequently exceeded 10 μg/L in this home despite removal efficiencies above 94.8%.

In a home with a very long LSL tested to 200% capacity, the duplicate POUs always reduced water lead levels <5 μg/L except for 3 (*n* = 40) samples. Under regular use by consumers in the NOLA and ELA residential homes, the POUs always reduced lead below 1 μg/L.

In waters with relatively high iron the filters clogged quickly which frustrated consumers. The reduction in the practical filter capacity can significantly increase the treatment costs of POUs, and even make bottled water more cost-effective and less burdensome for lead remediation in some circumstances. Clogging was not a significant problem in NOLA where iron was relatively low (median <5 μg/L, maximum 60 μg/L) but was a major concern in ELA where the median iron level concentration was relatively high (median 383 μg/L, maximum 19,700 μg/L).

## Supplementary Material



## Notes

Unoccupied Home Rig Schematic; Unoccupied Home Rig Pictures; Home with Sustained Lead (Home A)–particulate lead (concentration and percentage); Home with Disturbed LSL (Home B)–particulate lead (concentration and percentage); Filter Clogging for 3 faucet filter brands; Residential unfiltered and filtered water data in Enterprise (ELA) and New Orleans (NOLA), LA; and Residential sampling filter performance by home in Enterprise (ELA) and New Orleans (NOLA), LA (PDF)
