# Supplementary material for: Household Point-of-Use Faucet Filters for Lead Removal: Field Performance and User Experiences
Source: ACS ES T Water. 2025 May 9;5(6):3045–53. doi: 10.1021/acsestwater.4c01257 (PMC12172044; doi:10.1021/acsestwater.4c01257)
Supplement: Supplementary file 1 [file ew4c01257_si_001.pdf]

Household Point-of-Use Faucet Filters for Lead Removal: Field Performance and User Experiences

Jeannie M Purchase,[1] Chantaly Villalona,[1] Adrienne Katner,[2] Kelsey Pieper,[3] Marc Edwards*[1]
[1]Virginia Tech, Department of Civil and Environmental Engineering, Blacksburg, VA, 24061, USA
[2]Louisiana State University Health Science Center, Department of Environmental and Occupational Health Sciences, New Orleans, LA 70112, USA
[3]Northeastern University, Department of Civil and Environmental Engineering, Boston, MA 02115, USA

*Corresponding author: edwardsm@vt.edu; 407 Durham Hall, 1145 Perry Street Blacksburg, VA 24061

SUPPORTING INFORMATION

1. Figure S1: Unoccupied Home Rig Schematic
2. Figure S2: Unoccupied Home Rig Pictures
3. Figure S3: Home with Sustained Lead (Home A)– Particulate Lead (concentration and percentage)
4. Figure S4: Home with Disturbed LSL (Home B)– Particulate Lead (concentration and percentage)
5. Figure S5: Filter Clogging for 3 faucet filter brands
6. Table S1: Residential Unfiltered and Filtered water data in Enterprise (ELA) and New Orleans (NOLA), LA
7. Table S2: Residential Sampling filter performance by home in Enterprise (ELA) and New Orleans (NOLA), LA

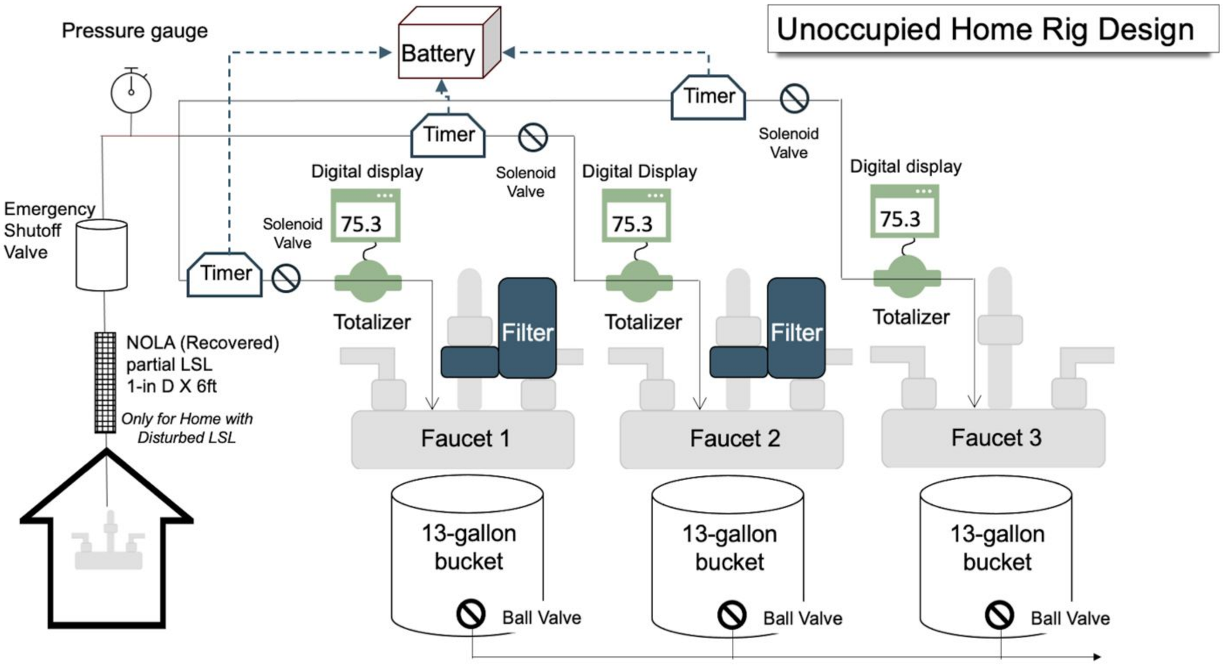

Figure S1. Unoccupied Home Rig Design

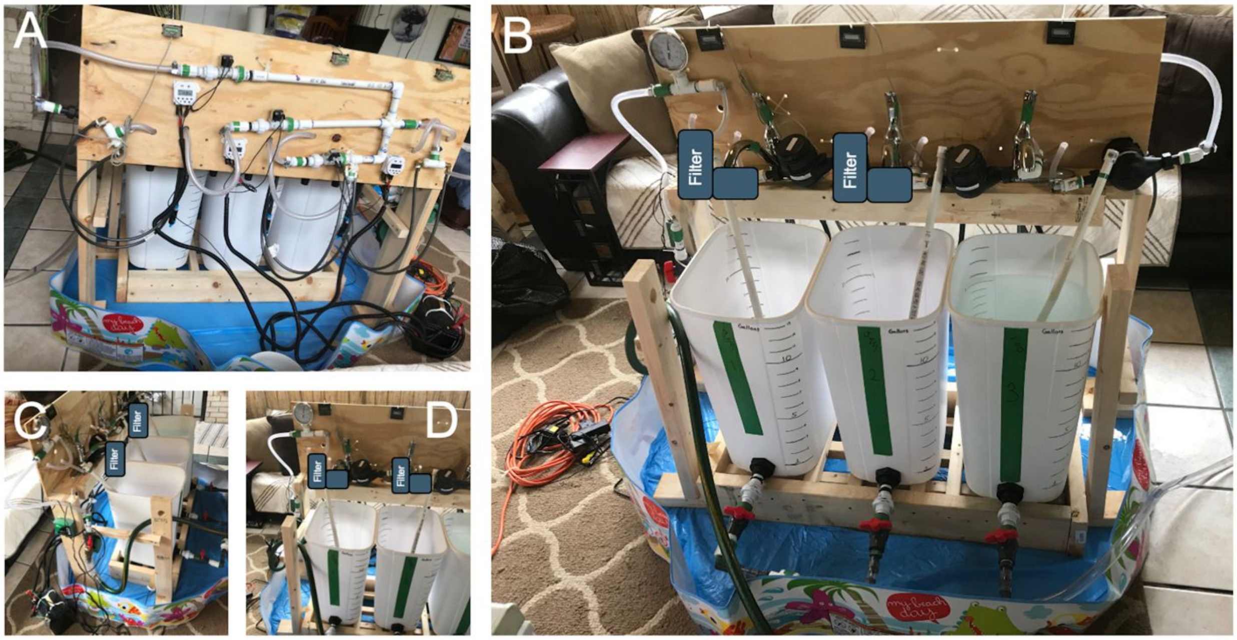

Figure S2. Unoccupied Home Rig Pictures

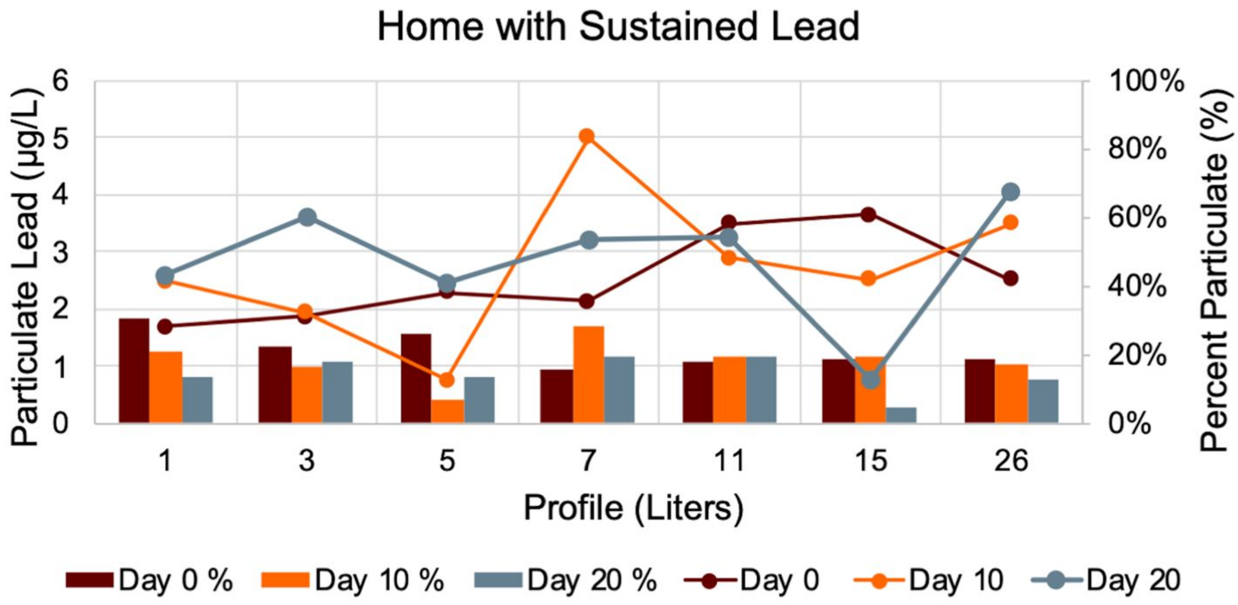

Figure S3. Home with Sustained Lead (Home A)– Particulate Lead (concentration and percentage)

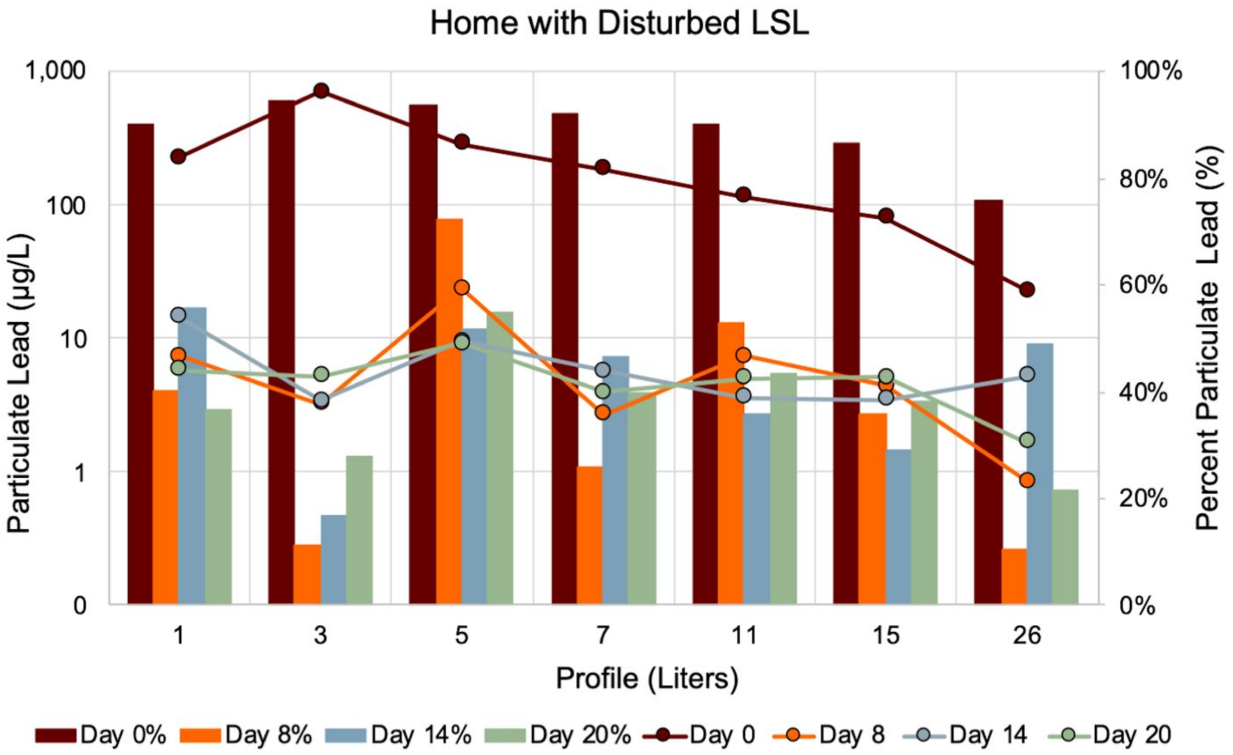

Figure S4. Home with Disturbed LSL (Home B) – Particulate Lead (concentration and percentage)

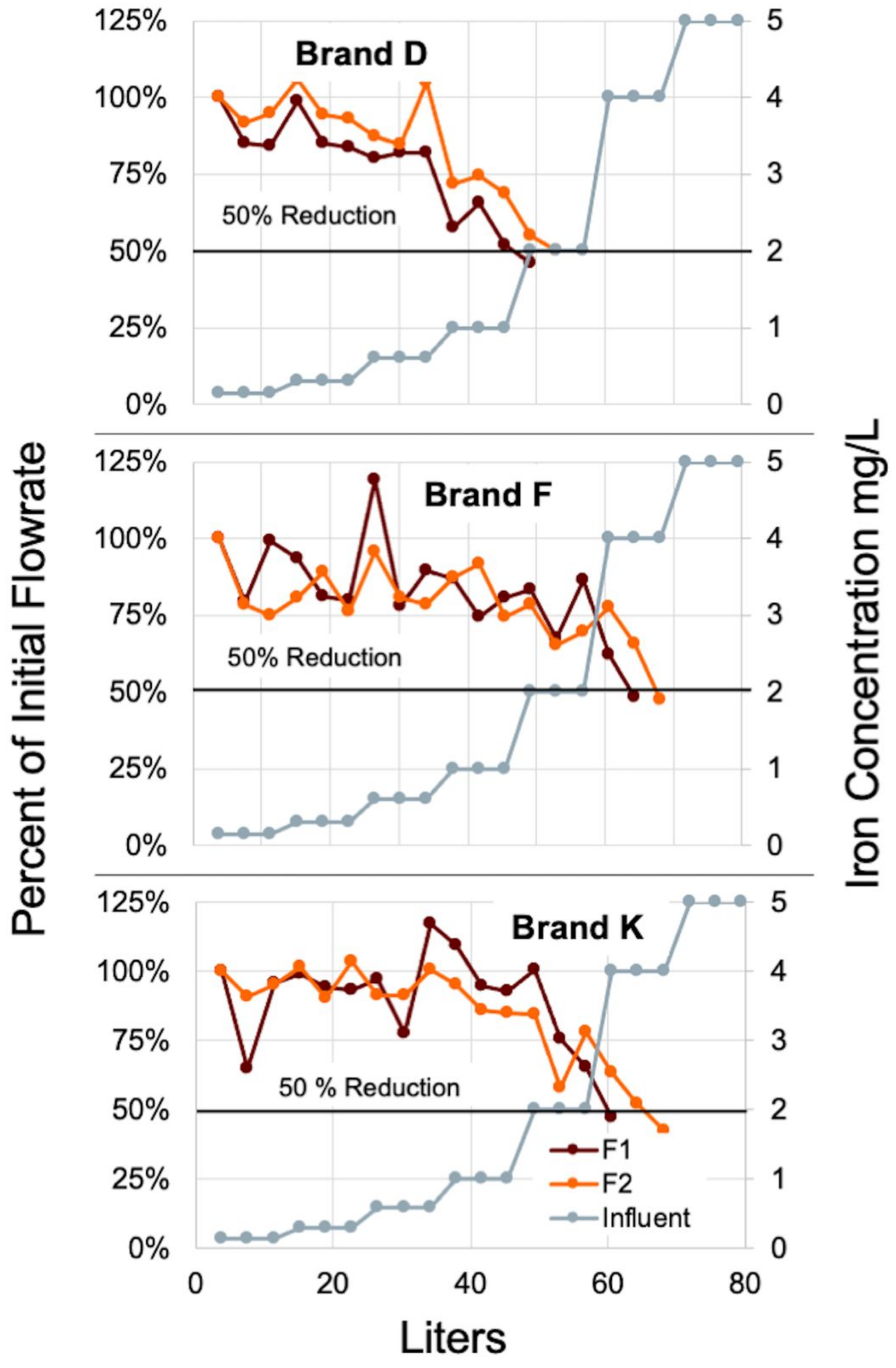

Figure S5. Filter Clogging for 3 faucet filter brands

Table S1. Residential Unfiltered and Filtered water data in Enterprise (ELA) and New Orleans (NOLA), LA

	Metal	Min (µg/L)	1st Qu. (µg/L)	Median (µg/L)	Mean (µg/L)	3rd Qu. (µg/L)	Max (µg/L)	n
ELA	Pb – UF	0.1	0.1	1.0	7.0	7.9	86.0	88
	Pb – F	0.1	0.1	0.1	0.2	0.1	2.9	88
	Fe – UF	15.0	179	383	762	587	19700	88
	Fe – F	5.0	5.0	5.0	8.8	5.0	171	88
	Mn – UF	5.6	67.2	106	142	162	917	87
	Mn – F	0.2	1.8	5.1	18.1	21.2	180	87
NOLA	Pb – UF	0.1	0.9	2.0	2.3	3.2	22.0	77
	Pb – F	0.1	0.1	0.1	0.1	0.1	0.8	77
	Fe – UF	5.0	5.0	5.0	7.6	7.1	57.3	77
	Fe – F	5.0	5.0	5.0	6.2	5.0	53.7	77
	Mn – UF	0.1	0.4	0.6	0.6	0.9	1.3	77
	Mn – F	0.1	0.1	0.2	0.6	0.2	19.1	77

Table S2. Residential Sampling filter performance by home in Enterprise (ELA) and New Orleans (NOLA), LA

Resident		Lead (Pb)			Iron (Fe)			Manganese (Mn)			Filter Life (Weeks)
		Unfiltered (µg/L)	Filtered (µg/L)	Avg Removal	Unfiltered (µg/L)	Filtered (µg/L)	Avg Removal	Unfiltered (µg/L)	Filtered (µg/L)	Avg Removal	
ELA	R1	<0.1 - 1.8	<0.1 - 0.2	90.9	33.9 - 1,150	<0.1 - 17.9	92	5.6 - 280	0.3 - 70.6	77.3	2,3
	R2	0.1 - 18.5	<0.1	99.3	42.0 - 384	<0.1 - 3.0	99.5	35.7 - 102	2.0 - 11.0	90.3	5
	R3	0.1 - 2.3	<0.1 - 0.2	79.7	62.3 - 1,230	0.5 - 6.7	98.6	25.1 - 376	1.6 - 11.3	82.3	6
	R4	<0.1 - 2.0	<0.1	100	15.0 - 2,650	<0.1 - 1.6	98.6	7.2 - 554	1.2 - 27.2	72.4	2,4,1
	R5	0.5 - 41.8	<0.1 - 0.1	98.3	89.4 - 1,210	<0.1 - 6.3	99.5	58.3 - 204	0.7 - 23.6	93.3	5,2
	R6	0.1 - 0.5	<0.1	100	377.0 - 3,270	3.9 - 8.1	98.7	83.1 - 860	2.8 - 43.7	77.4	5
	R7	10.6 - 38.6	<0.1 - 0.2	99.6	151.7 - 699	1.3 - 9.9	98.8	72.4 - 139	2.3 - 8. 0	93.8	6
	R8	0.2 - 86.0	<0.1 - 0.1	91.3	161 - 19,700	<0.1 - 171	99.2	28.7 - 917	0.2 - 25.2	96.2	1,3,2
	R9	<0.1 - 4.4	0.1 - 0.4	-	59.6 - 613	1.3 - 111. 1	91.9	136.0 - 260	99.4- 180.2	23.2	4
	R11	0.1- 10.3	<0.1 - 0.1	79.4	188 - 455	<0.1 - 6.9	98.8	42.9 - 146	2.2 - 89.1	44.7	6
	R12	0.1 - 1.8	<0.1	100	311- 836	<0.1 - 2.2	99.8	86.6 - 275	1.3 - 51.2	81.7	8
	R13	0.1 - 12.2	<0.1 - 0.2	99.7	159- 453	<0.1 - 6.9	99.5	45.1 - 202	1.5 - 5.7	96.2	8
	R14	<0.1 - 4.2	<0.1 - 0.1	99.7	155 - 632	<0.1 - 8.5	98.9	22.8 - 161	0.8 - 2.7	98.1	4
NOLA	R1	1.4 - 2.8	<0.1 - 0.4	97.4	<0.1 - 33.7	<0.1 - 53.7	-	0.6 - 1.3	0.1 - 1.1	54.3	9
	R2	<0.1 - 6.4	<0.1	100	<0.1 - 1.9	<0.1 - 29.5	-	0.2 - 0.8	0.1 - 0.4	74.9	10
	R3	3.3 - 22.0	<0.1 - 0.1	99.6	<0.1 - 1.7	<0.1 - 0.2	100	0.1	0.1 - 0.2	-	10
	R6	0.6 - 2.7	<0.1 - 0.3	97.5	<0.1 - 57.3	<0.1 - 2.5	96.2	0.3 - 0.7	0.2 - 0.5	55	12
	R11	0.9 - 1.0	<0.1 - 0.1	98.4	2.8 - 7.0	2.1 - 5.2	25.1	0.5 - 1.1	0.1 - 0.3	74.9	5
	R12	1.5- 3.2	<0.1 - 0.1	99.2	0.1 - 27.4	3.1 - 9.8	45.8	0.5 - 1.3	0.1 - 1.2	65.8	8
	R13	0.1 - 1.9	<0.1	94.2	6.6 - 56.4	4.7 - 5.7	28.6	0.4 - 2.3	0.2 - 0.7	45.5	5
	R14	2.7 - 6.1	<0.1 - 0.8	96.6	2.4 - 9.9	3.5 - 7.5	7.6	0.4 - 1.1	0.1 - 19.1	-	12
